# Factors Affecting Patient Decision-Making Regarding Midshaft Clavicle Fracture Treatment

**DOI:** 10.7759/cureus.10505

**Published:** 2020-09-17

**Authors:** Giancarlo Medina Perez, Megan M Tran, Christopher McDonald, Ryan O'Donnell, Aristides I Cruz, Jr.

**Affiliations:** 1 Department of Orthopaedic Surgery, Warren Alpert Medical School, Brown University, Providence, USA

**Keywords:** shared decision-making, trauma, clavicle fractures, radiographs, patient preferences

## Abstract

Introduction

Midshaft clavicle fractures are a common problem encountered by orthopedic surgeons. There remains debate between non-surgical and surgical treatment options for certain midshaft clavicle fractures. Due to the lack of a clear treatment strategy, this presents an opportunity for shared decision-making, which has been shown to be important to patients.

Methods

A 19-question survey was created encompassing basic demographic information, then taking respondents through a simulation of a midshaft clavicle fracture patient encounter. Subjects were subsequently asked their preferred treatment choice as well as shared decision-making preferences for the simulated encounter. A pilot study was performed with medical students from our home institution to assess study sample size. The survey was then distributed through an online software platform (Amazon Mechanical Turk). Statistical analysis was performed using STATA, Microsoft Excel, and Qualtrics.

Results

253 subjects responded to the online survey. Over 70% of respondents had no to minimal knowledge of clavicle fractures and potential medical interventions/treatments. 67.6% of respondents preferred shared decision-making, over autonomous or paternalistic models. 45.5% of respondents wanted additional time outside the physician-patient consultation before making a treatment decision. A majority of the respondents who selected surgery (44.3%; 43/97) and no surgery (69.9%; 109/156), based their decisions on outcomes data provided in the simulation alone. There was no statistically significant relationship between income, race/ethnicity, education level, work status, sex, or type of visual fracture representation (i.e., radiograph vs. cartoon image) and treatment decision (p>0.05). Younger age (p=0.007) and being married (p=0.001) were associated with increased likelihood to select surgery as the treatment decision.

Conclusion

Most respondents had no-to-minimal knowledge about clavicle fractures, placed a high value in shared decision-making for midshaft clavicle fractures, and prioritized outcomes data in making treatment decisions. Younger age and marital status may increase the likelihood of a patient selecting to proceed with surgery over non-operative treatment.

## Introduction

Clavicle fractures account for 2.6%-10% of all fractures [1-3] and midshaft (middle-third) fractures are the most common type, accounting for approximately 75%-80% of all clavicle fractures [1]. The ideal treatment for midshaft clavicle fractures remains controversial. A recent study demonstrated higher nonunion rates and functional deficits after nonsurgical treatment of certain midshaft clavicle fractures when compared to open reduction and internal fixation [4]. However, another study showed that the chances of sustaining a surgery-related complication after midshaft clavicle fracture fixation may be as high as 20% [5]. In a recent Cochrane Review consisting of 14 studies involving 1469 participants with acute midshaft clavicle fractures, Lenza et al. concluded that surgery may not yield superior benefit over conservative treatment in terms of function, pain, and quality of life. However, many of the studies included were of low-quality evidence [6].

Given the current lack of definitive treatment recommendations for midshaft clavicle fractures, the choice of treatment may be preference-sensitive in many cases and may be suitable for the use of a shared decision-making (SDM) approach. In SDM models, patients discuss treatment options closely with their provider to arrive at a mutually agreed upon treatment plan that takes into account overall patient values and goals [7]. Recent studies have supported the idea that patients prefer SDM over paternalistic or informed/autonomous models when making treatment decisions across both non-surgical and surgical clinical settings including orthopedics [8,9]. Despite the amount that is known on SDM, there is little literature specifically studying SDM for elective midshaft clavicle fracture surgery. Available studies have looked at the prevalence of SDM in midshaft clavicle fracture treatment discussions and suggest use of SDM may serve as a predictor of patient overall satisfaction [10,11]. However, no studies to our knowledge have analyzed the specific factors affecting treatment decision-making for midshaft clavicle fractures.

This study aimed to assess the factors that are important in influencing patients’ decisions to proceed with surgical versus non-surgical treatment for midshaft clavicle fractures and to highlight patient preferences in physician-patient discussions regarding treatment of adult midshaft clavicle fractures.

## Materials and methods

Study design

We developed an online survey using Qualtrics software, Version XM (Qualtrics, Provo, UT) describing a simulation of a midshaft clavicle fracture case. Demographic questions collected information on respondents’ age, race/ethnicity, sex, education level, work status, marital status, and income. Subsequently, participants were presented with background text on midshaft clavicle fractures and potential treatment options. The background highlighted the benefits of both surgical and non-surgical approaches for midshaft clavicle fractures. This component of the study was included to simulate a midshaft clavicle fracture patient-physician encounter. Information covered in the text included the definition of a clavicle fracture, treatment options available, risks and benefits of different treatment options, and long-term clinical outcomes based on currently available literature [4,12,13]. The text shown to respondents reads as follows:

"You will now enter the Clavicle Fracture specific portion of this survey. Before answering the questions, please read the prompt below: 

The clavicle or “collar bone” is one of the bones of the shoulder. Clavicle fractures (broken bones) are a commonly encountered injury. They usually happen after a traumatic event such as a fall or collision during a sporting event or an accident. 

Most of these fractures will heal on their own without surgery. In some cases, surgery is the preferred treatment, although surgery can have complications including bothersome surgical implants, infection, and damage to the surrounding structures (such as nerves and blood vessels). Some studies show that the chances of sustaining a surgery-related complication can be as high as 20%. 

Without surgery, the chances of not healing or healing incorrectly have been found to be somewhere between 2% and 15% (85%-98% heal correctly). Although relatively rare, the consequences of not healing or healing incorrectly include long-term pain or difficulty using the involved arm. Patients may also notice a prominence on the collar bone where fracture has healed when treated without surgery. In some cases, future surgery is needed to correct the collar bone that did not heal or healed incorrectly. 

There have been many studies looking at the results after treatment of clavicle fractures in adults. Surgery has been shown to decrease the rate of not healing or healing incorrectly but increases the risk of having a surgery-related complication. Overall, these studies show that, while there may be some short-term advantages to surgical treatment, the long-term (at least two years after treatment) outcomes are the same or similar between patients who choose surgery vs. those who do not choose surgery to treat their clavicle fracture."

**Figure 1:**
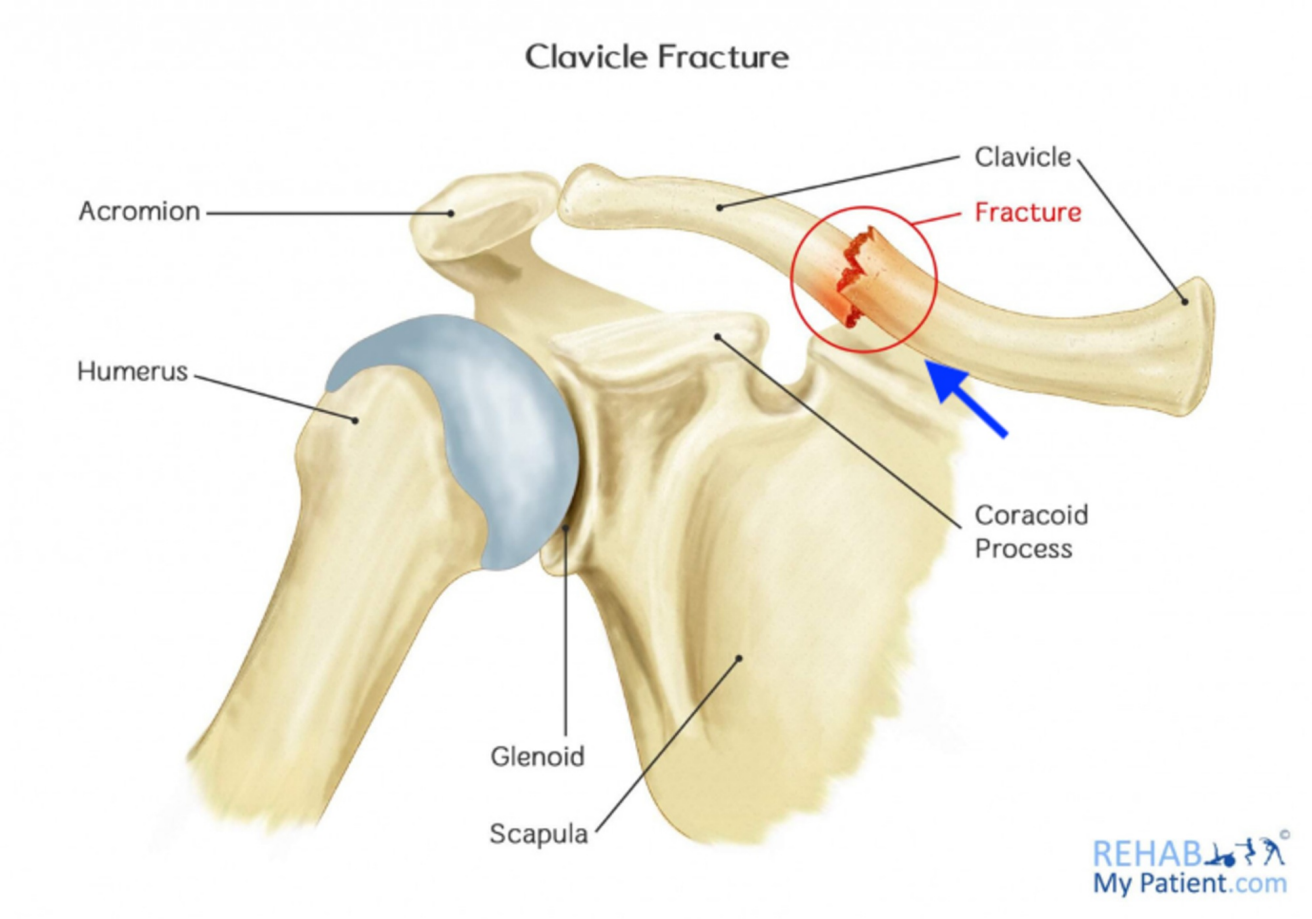
Clavicle fracture cartoon Ref. [14]

**Figure 2:**
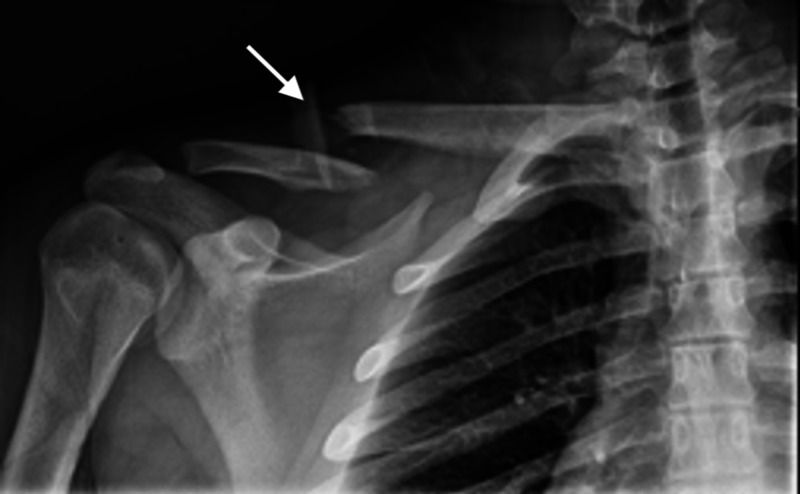
Clavicle fracture radiograph

We utilized Qualtrics software to randomly assign participants into three conditions: (1) No image of a midshaft clavicle fracture, (2) Cartoon schematic image of a midshaft clavicle fracture, (3) Radiographic image of a midshaft clavicle fracture. This participant distribution allowed us to study the influence of imaging in the patient decision-making process for midshaft clavicle fractures. For the “No image” group, only the background text was presented. For the “Cartoon image” group, the background text and a cartoon schematic depicting a clavicle fracture were presented (Figure 1). For the “Radiographic image” group, the background text and a radiograph depicting a midshaft clavicle fracture were presented (Figure 2). The “Cartoon image” group was included to reflect office-based posters and other material that may be shared by a physician during a midshaft clavicle fracture treatment discussion, while the “Radiographic image” group was included to reflect actual radiographic images that may be presented and discussed by a physician during a midshaft clavicle fracture treatment discussion.

Respondents were then asked to answer 9 to 12 questions under the simulation that they had sustained a midshaft clavicle fracture injury. The number of questions varied because some questions were prompted based on previous responses. Topics covered in the questions included previous knowledge regarding clavicle fractures, previous knowledge regarding medical interventions/treatments for clavicle fractures, preferred amount and type of information needed to make a clavicle fracture treatment decision, amount of time the participant would like to have before making a clavicle fracture treatment decision, preferred level and type of physician involvement in clavicle fracture treatment decision, rationale behind clavicle fracture treatment decision, and factors influencing confidence in clavicle fracture treatment decisions. 

A pilot test was conducted with first-year medical school students at the authors’ institution to optimize question selection and phrasing, as well as identify any administrative issues relating to survey distribution prior to the official study.

Study size

An a priori power analysis was conducted to compute a required sample size utilizing an effect size of 0.3 and a power (1-Beta error probability) of 0.95. This power analysis was based on the pilot study data for the relationship between clavicle fracture treatment decision (surgery versus no surgery) and imaging condition (no image, X-ray, or cartoon). The power calculation estimated a total of 172 participants to achieve adequate power.

Setting

The online survey was distributed during a three-day period (March 13th, 2020-March 16th, 2020) and was closed once 253 responses had been successfully received and verified for completion. 

Participants

The main study included a total of 253 participants. Eligibility criteria included being >18 years old and being located within the United States. To recruit participants for the study, we used Amazon’s Mechanical Turk (MTurk) platform. This platform contains thousands of active participants that are available to complete different tasks, including marketing and academic research surveys. On the MTurk platform, the survey was presented broadly as a survey related to clavicle fractures and MTurk members were allowed to opt in to participate in the survey anonymously if they met the eligibility criteria. Once the MTurk member opted in to participate, the survey link was sent to them. Each participant was compensated US $1 for their time in completing the survey. After survey completion, each respondent received a survey code for payment.

Data analysis/statistical methods

To analyze the data, we first used descriptive statistics to analyze the results of each of the questions of the survey. Chi-square analysis was then used to compare the distribution of categorical survey results. For cases in which the expected value or frequency was less than 5, Fisher’s exact test was used. The relationship between clavicle fracture treatment decision and variables including demographic features, imaging condition (no image, X-ray, or cartoon), and previous knowledge of clavicle fractures was evaluated. Multivariate logistic regression was used to assess the relationship between demographic factors (i.e., age, sex, and relationship status) and surgical treatment decision. Statistical significance was set at 0.05. Statistical analyses were performed with Stata Statistical Software: Release 16 (StataCorp, College Station, TX, USA), Qualtrics software, Version XM (Qualtrics, Provo, UT), and Microsoft Excel 2013 (Microsoft Corp, Redmond, WA, USA).

Validation evaluation

A pilot study was conducted to refine the questionnaire prior to the main study. No formal validation was performed for the questionnaire or for the curated write-up. All authors reviewed each component of the survey, data analysis, and manuscript for quality control purposes. Author consensus was reached before submitting the questionnaire.

## Results

Pilot study

The pilot study’s response rate was 36.3% (53/146). The mean age of respondents was 25 years and all respondents were first-year medical students. 58.5% (31/53) of respondents believed that the information provided was sufficient to make a decision on a preferred treatment for their clavicle fracture. All respondents indicated that they would want a doctor to be involved in their decision-making process: 98.1% (52/53) preferred to make a decision with their doctor and 1.9% (1/53) preferred their doctor to make the decision for them. In the group that chose a shared decision-making approach, 82.7% (43/52) of respondents preferred that their doctor gave recommendations on how to proceed before they made their final treatment decision, while 17.3% (9/52) wanted their doctor to only provide information and answer questions before they made the final treatment decision. Regarding treatment decisions, 41.5% (22/53) of respondents decided no surgery, 47.2% (24/53) were not sure, and 11.3% (6/53) wanted surgery. When grouping respondents based on their assigned imaging conditions, we found that 0% (0/19) of the “No image”, 7% (1/14) of the “Cartoon image”, and 25% (5/20) of the “Radiograph image” groups chose surgery as their preferred treatment for clavicle fractures. However, there was no statistically significant relationship between imaging condition and preferred treatment for midshaft clavicle fractures (p=0.132) (Table 1).

**Table 1:** Pilot study

Survey Results: Preferred Treatment for Clavicle Fracture								
Categories	Text Only (n=19)		Text + Cartoon (n=14)		Text + Radiograph (n=20)		Total (n=53)	
	No.	%	No.	%	No.	%	No.	%
No surgery	10	52.6	5	35.8	7	35.0	22	41.5
Not sure	9	47.4	8	57.1	8	40.0	24	47.2
Surgery	0	0.0	1	7.1	5	25.0	6	11.3

Main study 

**Figure 3:**
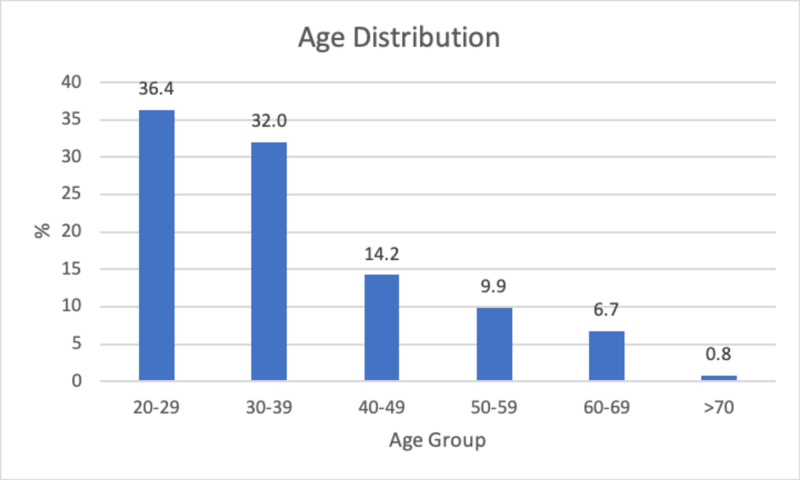
Age distribution

In the main study, a total of 253 responses were collected. The mean and median age of the respondents were 37 and 33, respectively (range 20-85 years). Figure 3 displays the age distribution of respondents. Tables 2-4 display the race/ethnicity, sex, and marital status of respondents. Tables 5-7 display the education level, work status, and annual household income of respondents.

**Table 2:** Race/ethnicity

Survey Results: Race/Ethnicity		
Categories	Total (n=253)	
	No.	%
American Indian or Alaska Native	1	0.4
Asian	14	5.5
Black or African American	24	9.5
Hispanic or Latino	25	9.9
Other	3	1.2
White	186	73.5

**Table 3:** Sex

Survey Results: Sex		
Categories	Total (n=253)	
	No.	%
Female	98	38.7
Male	155	61.3
Other	0	0.0

**Table 4:** Marital status

Survey Results: Marital Status		
Categories	Total (n=253)	
	No.	%
Divorced	13	5.1
Married or domestic partnership	134	53.0
Separated	4	1.6
Single, never married	100	39.5
Widowed	2	0.8

**Table 5:** Education level

Survey Results: Education Level		
Categories	Total (n=253)	
	No.	%
Associate degree	21	8.3
Bachelor's degree	129	51.0
High school graduate, diploma, or the equivalent	18	7.1
Master's degree	28	11.0
Professional degree or Doctorate degree (e.g. MD, JD, PhD)	5	2.0
Some college	42	16.6
Some high school	2	0.8
Trade/technical/vocational training	8	3.2

**Table 6:** Work status

Survey Results: Work Status		
Categories	Total (n=253)	
	No.	%
Employed	191	75.5
Out of work and looking for work	8	3.2
Out of work but not currently looking for work	8	3.2
Retired	6	2.4
Self-employed	35	13.8
Student	5	2.0

**Table 7:** Annual household income

Survey Results: Annual Household Income		
Categories	Total (n=253)	
	No.	%
Less than $25,000	35	13.8
$25,000 - $49,000	71	28.1
$50,000 - $99,000	101	39.9
$100,000 - $149,000	32	12.7
$150,000 - $199,000	11	4.3
$200,000 - $250,000	2	0.8
More than $250,000	1	0.4

Clavicle fracture-related knowledge

The majority of respondents had no (31.6%; 80/253) to minimal (38.7%; 98/253) knowledge of clavicle fractures prior to the study, while 24.9% (63/253) had a moderate amount of knowledge and 4.7% (12/253) had a high amount of knowledge. Similarly, a majority of the respondents had no (38.3%; 97/253) to minimal (36%; 91/253) knowledge of potential medical interventions/treatments available for clavicle fractures, while 21.7% (55/253) had a moderate amount of knowledge and 4% (10/253) had a high amount of knowledge.

Patient preferences

79.4% (201/253) of respondents believed that the information provided was sufficient to make a decision on a preferred treatment for their clavicle fracture. The 20.6% (52/253) who wanted more information would have liked more specifics about the severity of the clavicle fracture (32.1%; 35/109), more information on benefits and potential complications of surgery versus no surgery (28.4%; 31/109), more academic research or internet sources related to the topic (19.3%; 21/109), and/or a second opinion from another physician (18.4%; 20/109). Regarding time preferences, most respondents (54.5%; 138/253) indicated they were ready to make a decision, 22.1% (56/253) wanted some hours before deciding, 18.6% (47/253) wanted some days before deciding, and 4.7% (12/253) wanted more than a week before deciding. When asked about preferred physician involvement in the treatment decision, most respondents (82.2%; 208/253) indicated that they would seek some involvement from their physician, while 17.8% (45/253) wanted to make the decision on their own with no involvement from a doctor. Out of the respondents preferring some involvement from their physician, 67.6% (171/253) preferred to make a shared decision with their doctor and 14.6% (37/253) preferred their physicians to make the decision for them given their medical knowledge. Of those who elected for shared decision-making, 60.8% (104/171) wanted their physicians to give their recommendation on how to proceed, while 39.2% (67/171) only wanted their physicians to provide information and answer their questions before they made their own final treatment decision.

Treatment decision and rationale

When asked for a treatment decision, 61.6% (156/253) of respondents chose no surgery and 38.4% (97/253) chose surgery. For those who chose no surgery, 69.9% (109/156) based their decision on the information that was provided in the survey, 12.2% (19/156) did not want to undergo surgery no matter what, 10.9% (17/156) based their decision on prior knowledge/experiences regarding surgery, and 6.4% (10/156) felt they needed more information. For the respondents who selected surgery, 44.3% (43/97) determined the data convinced them, 29.9% (29/97) based their decision on the image of the fracture presented, and 25.8% (25/97) believed surgery is always the best option. 61.7% (156/253) of respondents felt moderate confidence in their treatment decision, followed by 31.6% (80/253) who felt high confidence and 6.7% (17/253) who felt low confidence. Those who felt low confidence would mostly want further discussion with their doctor (42.4%; 14/33) to increase their confidence in the treatment decision. Other information that some of these respondents would want include more information on potential complications of surgery vs. no surgery (18.2%; 6/33), more information on healing time of surgery vs. no surgery (18.2%; 6/33), and more time to conduct personal research online (18.2%; 6/33).

Relationship between clavicle fracture treatment decision and other factors

There was no association between the image conditions (i.e. “No image”, “Cartoon image”, and “Radiographic image”) and preferred treatment with the provided “No surgery” and “Surgery” options (p=0.623). We also analyzed the association between demographic variables and preferred treatment decision. To analyze the relationship between age and treatment decision, the sample was split at 33 years of age based on the median age of the overall group. For respondents less than 33 years, 51.2% (62/121) chose no surgery and 48.8% (59/121) selected surgery. For respondents greater than or equal to 33 years of age, 71.2% (94/132) selected no surgery and 28.8% (38/132) chose surgery. The difference in treatment decision based on age was statistically significant (p=0.001). There was also a statistically significant relationship between sex and treatment decision, with 30.6% (30/98) of female-identifying respondents selecting surgery compared to 43.2% (67/155) of male-identifying respondents selecting surgery (p=0.044). Lastly, there was a statistically significant relationship between respondents who were married versus not married and treatment decision (p=0.006). Among married respondents, 46.3% (62/134) chose surgery over no surgery, while 29.4% (35/119) of non-married respondents selected surgery over no surgery.

Multivariate logistic regression analysis revealed that the relationship between sex and clavicle fracture treatment decision was not significant (p=0.067). The relationship between treatment decision and age, as well as marital status, remained significant with p-values of 0.007 and 0.001, respectively. Married respondents had 2.57 higher odds of selecting surgery compared to unmarried respondents. With a beta coefficient of -0.03, for every additional year of age, there was a decreased likelihood for a respondent to choose surgery as their preferred treatment option (Table 8). Lastly, when running the same regression looking at the median age of 33, respondents under 33 years of age had 2.50 higher odds of selecting surgery compared to respondents 33 years of age and older (p=0.001). 

**Table 8:** Multivariate logistic regression for demographic factors associated with clavicle fracture treatment decision Note: The pseudo R^2^ value of this model was 0.0633.

Factor	Odds ratio value	Lower limit	Upper limit	P-value
Age	0.97	0.94	0.99	0.007*
Male	1.70	0.96	3.00	0.067
Married	2.55	1.47	4.43	0.001*

## Discussion

The results of this study highlight the importance of the physician-patient conversation in informing the treatment decisions regarding midshaft clavicle fractures. In the current study, about 70% of the respondents had no (31.6%; 80/253) to minimal (38.7%; 98/253) knowledge of clavicle fractures and almost 75% had no (38.3%; 97/253) to minimal (36%; 91/253) knowledge of potential medical interventions/treatments. The majority of respondents preferred a shared decision-making (SDM) approach over both no physician involvement and a physician-centered decision-making model when making clavicle fracture treatment decisions.

In the current study, 67.6% of participants reported that they would like to make a shared decision with their doctor. This is comparable with the 77.8% rate of preferred SDM for current health conditions and 65.1% for chest pain reported in a 2007 study examining patient roles in treatment decisions [8]. Furthermore, our findings were consistent with Woltz et al. who found a mean score of 74/100 for patients’ perceived degree of SDM in physician-patient consultations specifically discussing midshaft clavicle fractures [11]. Previous studies have supported that more patient involvement in the treatment decisions may be positively related to quality of life and overall satisfaction with treatment [10,15]. Therefore, the continued and expanded incorporation of SDM models or decision aids into clinical practice for midshaft clavicle fractures may be conducive to a better overall patient experience.

The preferred SDM scenario for a majority of the respondents who preferred an SDM model was one where the physician provided a specific treatment recommendation, followed by the patient making their own final treatment recommendation. Analyzing SDM use in orthopedic surgery specifically, Lindsay et al. found that outside of the treatment decision, patients would want to be most involved in surgery scheduling decisions and post-operative forms of care and least involved in the more technical aspects of surgery such as determining incision sizes [16]. Despite the high percentage of respondents who prefer an SDM approach, our survey results suggest that the percentage of individuals who prefer to make their treatment decision without any input from their physician was substantial at 17.8%. This rate of preference for autonomous decision-making was much higher than rates reported in previous studies [8,17]. By providing information on clavicle fractures and treatment options within the survey, respondents may have felt more empowered to make a treatment decision with minimal physician involvement. This may be analogous to providing curated, written material on common diagnoses to help inform patients in the office setting.

While a majority of respondents in our survey (54.5%; 138/253) were ready to make a treatment decision, 45.5% (115/253) would have liked more time before making a decision. This finding indicates that a substantial number of patients may not be ready to make a decision during their conversation with a physician and may need additional time before deciding to proceed with surgery versus no surgery. These findings are consistent with Andersen et al.’s study which found that patients undergoing lumbar disc herniation surgery wanted more time to process information given before making a treatment decision [18]. Kihlstrom et al. found that patients who underwent operative treatment for clavicle fractures in the acute stage did so after a median of five days [19]. Therefore, even in patients where their preferred treatment option is surgery, additional time should be allowed for patients to make a final treatment decision.

We did not find a statistically significant relationship between income, race/ethnicity, education level, work status, or sex and treatment decisions. The finding on income is consistent with Skinner et al.’s finding that there were no differences based on income for Medicare enrollees undergoing total knee arthroplasty [20]. However, the finding on race differs from Skinner et al. and Chibnall et al. who found significant relationships between African American and Asian race and likelihood of undergoing total knee arthroplasty or low back surgery [20,21]. Additionally, in the field of oncology, Restrepo et al. found that race, income, insurance, and education level all had a statistically significant association with breast reconstruction rates [22]. It may be that the influence of the above demographic factors on treatment decisions is diagnosis and treatment specific and that findings for one diagnosis or condition does not necessarily predict decisions on other diagnoses. 

The type of image shown did not influence participants’ treatment decisions. When respondents were asked about the rationale behind their treatment decision, a majority of the respondents who selected surgery (44.3%; 43/97) and no surgery (69.9%; 109/156) based their decision on the data presented and not on the type of image shown. While imaging may still have a role in patient treatment decisions, discussions on treatment outcomes may be of higher value to patients. It is also possible that individuals in our study may have viewed the image included in the simulation as a generic image and not their own. It is likely that in the real-world setting, an image of a patient’s actual injury would influence his/her treatment decisions differently.

The results of this study did demonstrate some demographic factors that are associated with clavicle fracture treatment preference. In our sample, those who were younger were more likely to choose surgery. The skew towards younger respondents choosing surgery may be because older adults tend to be more risk adverse and may choose a more conservative, nonsurgical approach [23]. Physicians should be aware of the relationship between treatment decision preference and age when interacting with older patients with predictive factors of nonunion (i.e., smoking history, a comminuted fracture, and highly displaced fractures), since surgical treatment options may be more favored in these cases due to fracture characteristics [24]. On the other hand, younger patients with fractures displaced less than 2 centimeters and with remaining bony contact should be informed that there is strong evidence in favor of non-operative treatments for their type of fracture [25,26]. Married individuals were also more likely to choose surgery to treat their clavicle fracture. This may be because married people are more likely to have health insurance than unmarried people, leading them to be more willing to undergo surgery, as the procedure would be covered by their insurance plan [27,28]. Unfortunately, we did not query the insurance status of individuals in our study so could not analyze this directly. Another possibility is that married people may have more post-operative support from spouses or children, which may make the surgical treatment option more attractive.

Limitations

This study has several limitations. Firstly, the United States MTurk worker population is not nationally representative of the population as a whole. Compared to the US population, MTurk workers tend to be younger, more educated, less likely to be married, more likely to be unemployed, and report lower personal incomes [29,30]. However, the higher incidence of clavicle fractures in individuals younger than 50 years and of male sex make this sample more reflective of the population likely to suffer from clavicle fractures [2,3,19]. More specifically, Kihlstrom et al. identified that 68% of the 2,422 clavicle fractures they studied occurred in males, with the largest group being in the 15- to 24-year-old age group [19].

Another limitation is that this study simulated a clavicle fracture clinical scenario. Individuals who experience the physical pain of a clavicle fracture may respond differently to questions that assess the importance of various factors in treatment decision-making. It is difficult to predict how the emotional impact of an injury may impact patient decision-making. However, our results support that within an SDM model, patients will lean on clinical data around risks and benefits of the surgical procedure for purposes of making a treatment decision. This indicates that the role of the provider in SDM protocol should be to provide as much data and information on risks and benefits, regardless of the patient’s desire to return early to work or the pain they are undergoing due to the fracture. 

An additional limitation to the study was the fact that in a real clinical scenario physicians are likely to present the radiograph to the patient and it is unlikely that a patient will receive no image or a cartoon image. However, in order to assess the relationship between imaging and clavicle fracture treatment decision, the authors determined that a randomization of respondents into different imaging conditions would be the most appropriate way to study this relationship.

Lastly, although the clavicle fracture information text presented to all respondents was the same, the type of image presented to respondents was different and could have influenced the survey respondents’ treatment choices. However, we specifically designed the survey in this manner to examine the influence of image type on treatment decision and we found no statistically significant difference between clavicle treatment decision and type of image presented.

## Conclusions

Literature indicates that there remains clinical equipoise between surgical and non-surgical management for midshaft clavicle fractures. Due to this, it is important to assess patients’ preferences regarding their role in midshaft clavicle fracture treatment decision-making and offer support through SDM. Demographic features, amount of patient involvement in treatment decisions, time before making a treatment decision and imaging may affect the decision-making process for patients. It is hoped that this study’s findings will help physicians better understand several factors that may influence midshaft clavicle fracture treatment decision-making and patient overall satisfaction. 

## Figures and Tables

**Figure 4:**
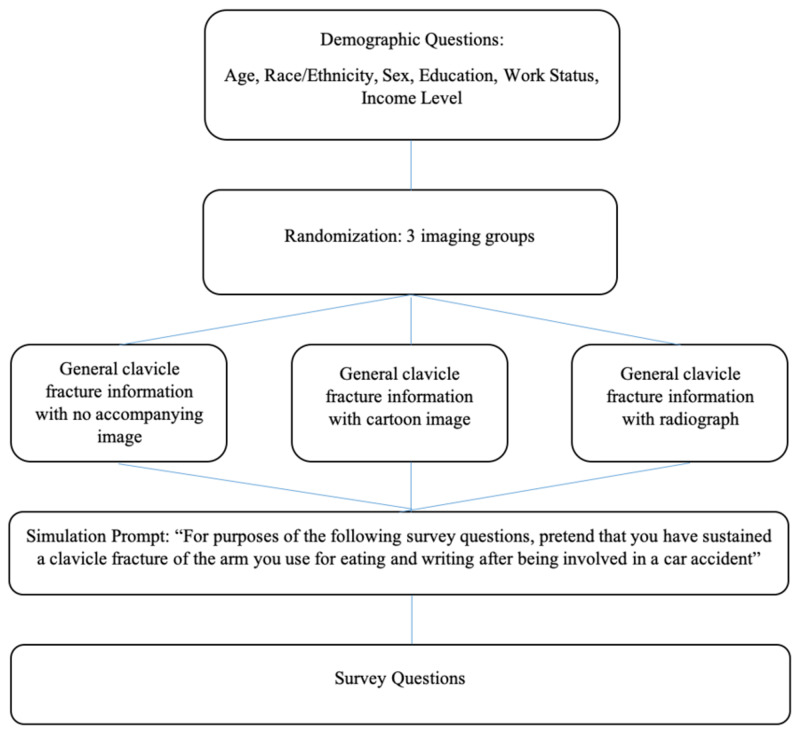
Clavicle fracture survey flow diagram

## Appendices

**Table 9:** Clavicle fracture survey questions

Questions	Answer Options
What is your age?	a. [Text Response – Number Only]: ______________________
What is your race/ethnicity?	a. American Indian or Alaska Native; b. Asian; c. Black or African American; d. Hispanic or Latino; e. Native Hawaiian or Other Pacific Islander; f. White; g. Other: __________
What is your sex?	a. Male; b. Female; c. Other: __________
What is the highest degree or level of school you have completed?	a. No schooling completed; b. Some high school; c. High school graduate, diploma, or the equivalent; d. Some college; e. Trade/technical/vocational training; f. Associate degree; g. Bachelor’s degree; h. Master’s degree; i. Professional degree or Doctorate degree (e.g. MD, JD, PhD)
What is your work status?	a. Out of work and looking for work; b. Out of work but not currently looking for work; c. Self-employed; d. Employed; e. Retired; f. Student
What is your marital status?	a. Single, never married; b. Married or domestic partnership; c. Widowed; d. Divorced; e. Separated
What was your annual household income this past year?	a. Less than $25,000; b. $25,000 - $49,000; c. $50,000 - $99,000; d. $100,000 - $149,000; e. $150,000 - $199,000; f. $200,000 - $250,000; g. More than $250,000
How much did you previously know regarding clavicle fractures?	a. No knowledge; b. Minimal amount of knowledge; c. Moderate amount of knowledge; d. High amount of knowledge
How much did you previously know regarding potential medical interventions/treatments for clavicle fractures?	a. No knowledge; b. Minimal amount of knowledge; c. Moderate amount of knowledge; d. High amount of knowledge
Do you feel the information provided at the beginning of the survey is sufficient to make a decision on a preferred treatment for your clavicle fracture?	a. Yes; b. No
[If “b. No” selected] What other type of information would you need to make a decision? Select all that apply.	a. More academic research or internet sources related to the topic; b. More specifics about severity of the clavicle fracture; c. More information on benefits and potential complications of surgery vs. no surgery; d. Second opinion from another physician; e. Other: _________________
Do you feel ready to make a decision on your preferred treatment for the clavicle fracture at this time?	a. Yes; b. No, I would need some hours before deciding (<24 hours); c. No, I would need some days before deciding (< 1 week); d. No, I would need more than a week before deciding (>1 week)
What would be your preferred level of involvement for the doctor in your treatment decision?	a. I would like to make the decision on my own with no involvement from my doctor; b. I would like to make a shared decision with my doctor; c. I would like my doctor to make the decision for me given his/her medical knowledge
[If “b. I would like to make a shared decision with my doctor” selected] What would you want the role of the doctor to be in that shared decision-making process?	a. I want my doctor to only provide information and answer my questions before I make the final treatment decision; b. I want my doctor to give me his/her recommendations on how I should proceed before I make the final treatment decision; c. Other: ______________________
After reading the above information about clavicle fractures, what would be your preferred treatment for your clavicle fracture?	a. No surgery; b. Surgery
[If “a. No surgery” selected] What is the main reason you chose no surgery as your preferred treatment?	a. I do not want to undergo surgery no matter what; b. Based on the information provided, no surgery seems like the better option; c. Given my prior knowledge/experiences, I would not want to have surgery; d. I need more information; e. Other: ______________________
[If “b. Surgery” selected] What is the main reason you chose surgery as your preferred treatment?	a. The image of the fracture convinced me to get it fixed; b. The data presented convinced me to get it fixed; c. I think surgery is always the best option; d. Other: ______________________
How confident are you in your treatment decision?	a. Low; b. Moderate; c. High
[If “a. Low” selected] What other type of information would you need to increase your confidence in your decision? Select all that apply.	a. Further discussion with my doctor; b. More information on potential complications of surgery vs. no surgery; c. Healing time of surgery vs. no surgery; d. Time to conduct personal research online; e. Other: ______________
